# Medical Toxicology vs. Emergency Medicine and Internal Medicine — Are We Really Full of Case Reports?

**DOI:** 10.1007/s13181-024-01031-y

**Published:** 2024-09-20

**Authors:** Jason B. Hack, Kevin F. O’Brien

**Affiliations:** 1 Department of Emergency Medicine, ECU Health, East Carolina University, 600 Moye Blvd, Greenville, NC 27834 USA; 2 Department of Public Health, Brody School of Medicine, East Carolina University, 600 Moye Blvd, Greenville, NC 27834 USA

**Keywords:** Case Report, Comparison, Publication Choices, Research, Literature Hierarchy

## Abstract

**Introduction:**

Case reports are perceived as having diminished value relative to other study designs. It has been said that medical toxicology (MT) is based largely upon case report literature and thought to be unique in this regard. We sought to quantify recent MT publication of case reports compared with top periodicals from emergency medicine (EM) and internal medicine (IM) journals.

**Methods:**

A retrospective review examined 5 years of articles in 6 U.S.-based medical journals–MT (*Journal of Medical Toxicology*, *Clinical Toxicology*), EM (*Annals of Emergency Medicine*, *Journal of Emergency Medicine*), and IM (*JAMA Internal Medicine*, *New England Journal of Medicine*) was performed using on-line resources. Every article in each issue was categorized into Case report vs. Research and Analysis articles vs. Excluded. “Case report” was defined as one (or *≤* 5) individual patients, one patient’s data, etc. Total articles per issue were reported after removing Excluded items.

**Results:**

Between 2018 and 2022, these 6 periodicals published 522 issues; with 2644 case reports; and 8246 total included articles. Comparison of MT case reports vs. EM revealed a significant difference and odds (Odds Ratio = 1.7, (95% CI: [1.49, 2.03], *p* < 0.001); MT compared with IM was not significantly different (Odds Ratio = 1.1, (95% CI: [0.96, 1.30], *p* = 0.150). The percent of case reports increased in the IM and EM journals compared with a relative decrease in the MT journals. Cumulative case report precents were consistently greater in EM and IM than in MT.

**Conclusion:**

In the past 5 years, MT journals published fewer and had a declining trend of case reports compared with leading EM and IM journals. Future research is needed to determine the effect on MT practice resulting from the diminished body of case report literature.

##  Introduction

Case reports, defined as “a formal summary of a unique patient and his or her illness, including the presenting signs and symptoms, diagnostic studies, treatment course and outcome” [1], have historically created the foundation for medical literature and provided the vehicle for dissemination of clinically relevant information [2]. In more modern times their value as a medical article was downgraded and it was fixed in a low-ranking position at the bottom of the accepted literature-evidence hierarchy [3, 4].

It has been said the practice of medical toxicology is based largely upon case report literature and thought to be unique among other specialties in this regard; “Your literature is all case reports.” (personal communication, ICU attending, Jan, 2023), [5]. While this statement’s veracity is debatable, a comparison of case report content in recently published top periodicals from other specialties has not been done and would provide valuable insights into the recent publishing trends within each field to objectively determine case report frequencies between medical concentrations.

We undertook this evaluation to clarify several issues: (1) identify the percentage of case reports published in leading medical toxicology (MT) periodicals annually and overall for the last 5 years, (2) compare the percentage of case reports published in MT journals annually and cumulatively over 5 years with leading journals from emergency medicine (EM) and internal medicine (IM), and (3) Determine and compare trends in publication of case reports over the past 5 years for these medical journals.

## Methods

This retrospective review examined 5 years of articles (2018–2022) in 6 U.S.-based medical journals -- MT (*Clinical Toxicology*, *Journal of Medical Toxicology*), EM (*Annals of Emergency Medicine*, *Journal of Emergency Medicine*) and IM (*New England Journal of Medicine*, *JAMA Internal Medicine*). Each article was reviewed by one unblinded reviewer (JBH), with no conflict of interest. Articles were reviewed online at publisher’s website or through the university’s medical library portal. The selection of the EM and IM journals was made based on several factors—a foundational specialty journal paired with one primarily with an on-line presence, high impact factor, and name recognition. This study was determined to be exempt by the East Carolina University and Medical Center Institutional Review Board.

Every article in each issue was reviewed individually. These were split a priori into groups where the article’s data was examined using a previously determined formalized categorical definition for case reports, research and analysis articles, and excluded articles.

### Category Definitions

#### Case Reports

Articles included in this category primarily fulfilled the definition ‘A detailed description of the experience of a single patient’ [6] we included cross-sectional articles [7] that were about individual patients at one point in time, or one patient’s data (their image, testing, examination findings, etc). These were generally firsthand encounters. We also included in this category small case series (*≤* 5 patients) to capture reports of grouped exposures within one event (e.g. “3 patients presented after eating castor beans…”). Broadly, this category included any article that reported a patient (or very small group of patients), where the focus was aimed at detailing a specific encounter, event, or finding.

#### Research and Analysis Articles

Included in this category was any article that reported research reports or any formal studies (poison center collections of data, groups of humans or animals, collections of humans (> 5) with an exposure, etc.); collections of people identified through a data set (e.g. a study searched a data base and only came up with few people it was considered research (“a literature review revealed 5 patients with…”)); if the small group of people discussed in paper presented at different times to one center it was considered research (“over the last 10 years, three patients presented with…”); population reviews. Also included in this category were no-patient articles: position statements, journal club, thoughtful discussions of a topic, literature review on a topic, abstracts from a conference, poison center annual reports, consensus statements, systemic reviews; “How To” articles (e.g. “we tried out this new device/new use of a device on one/few patient(s) and this is what we found…” [where the focus is on the intervention, not the patient]); etc. Broadly, this category included articles that examined and reported on a body of data (involving patients, literature or techniques) that was searched, researched, compiled, queried, analyzed, etc.

#### Excluded Articles

Included in this category were any editorials, reflections, perspectives, book reviews, acknowledgements, “comment on…”, in memoriam, laudatory articles about individuals, etc. Each letter to the editor of each journal was individually reviewed within the correspondence section — discussions of previously published articles including criticisms, corrections, and opinions were not counted; presentation and discussion of individual cases were considered ‘Case Reports’. Broadly, this category of excluded articles was everything else not previously included.

Total included articles in each issue or journal were the sum of Case Reports plus Research and Analysis articles after the removal of Excluded articles.

Statistical Methods employed: Article counts and proportions were used for descriptive purposes. Continency tables and Pearson chi-squares were used for inference with odds ratios as measures of association. Pearson Chi-squares and Wald tests were two tailed. Logistic regression was used to assess trends over years and compare trends across groups via interaction terms of year and journal. The 0.05 level of significance was used for all tests.

## Results

**Fig. 1:**
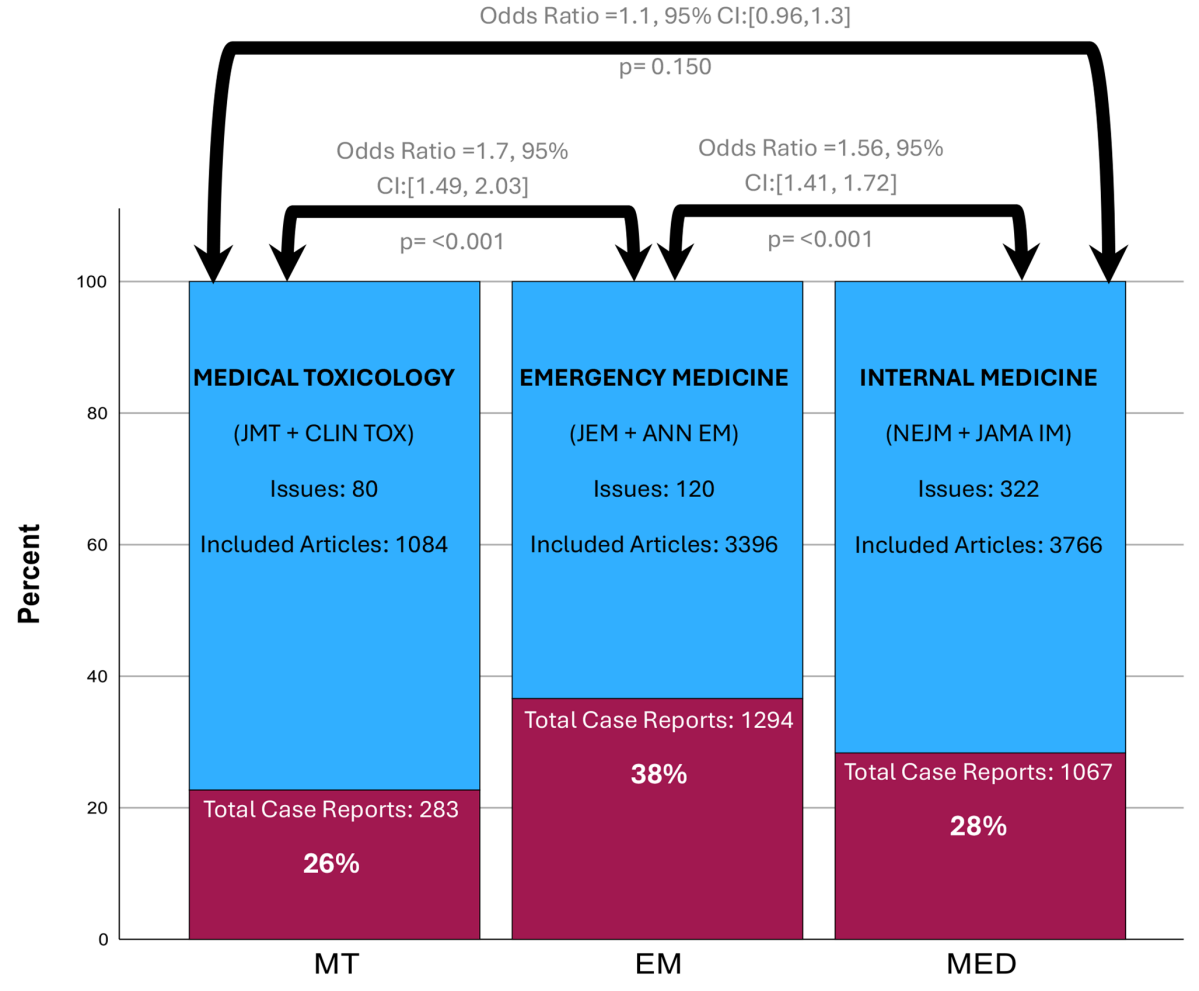
Percent of Case Reports in Each Specialty Journal Cumulative over 5 years (2018-2022). Pearson Chi-Square [2×2 table]

Between 2018 and 2022, these 6 periodicals published 522 issues (MT-80; EM-120; IM-322). There were 2,644 Case reports (MT-283; EM-1294; IM-1067); and 8,246 Total included articles (MT-1084; EM-3396; IM-3396). (Fig. 1).

Using an odds ratio as an effect measure to demonstrate the degree of difference and Pearson Chi-square statistics using the relevant 2 × 2 table to generate significance levels, we compared the specialties. Comparing cumulative MT case reports, (283; 26.1%) vs. EM (1294; 38.1%) revealed a significant difference between journal specialty and the odds of case reports (Odds Ratio = 1.7, (95% CI: [1.49, 2.03], *p* < 0.001); MT (283;26.1%) relative to IM (1067; 28.3%) revealed no significantly different association (Odds Ratio = 1.1, (95% CI: [0.96, 1.30], *p* = 0.150).

The percentage of cumulative case reports for each year was consistently greater in EM and IM than it was in the MT journals. Comparing trends in the percent of case reports, they were noted to have increased over the included 5 years both in the IM and EM journals while there was a relative decrease in the MT journals.

**Fig. 2:**
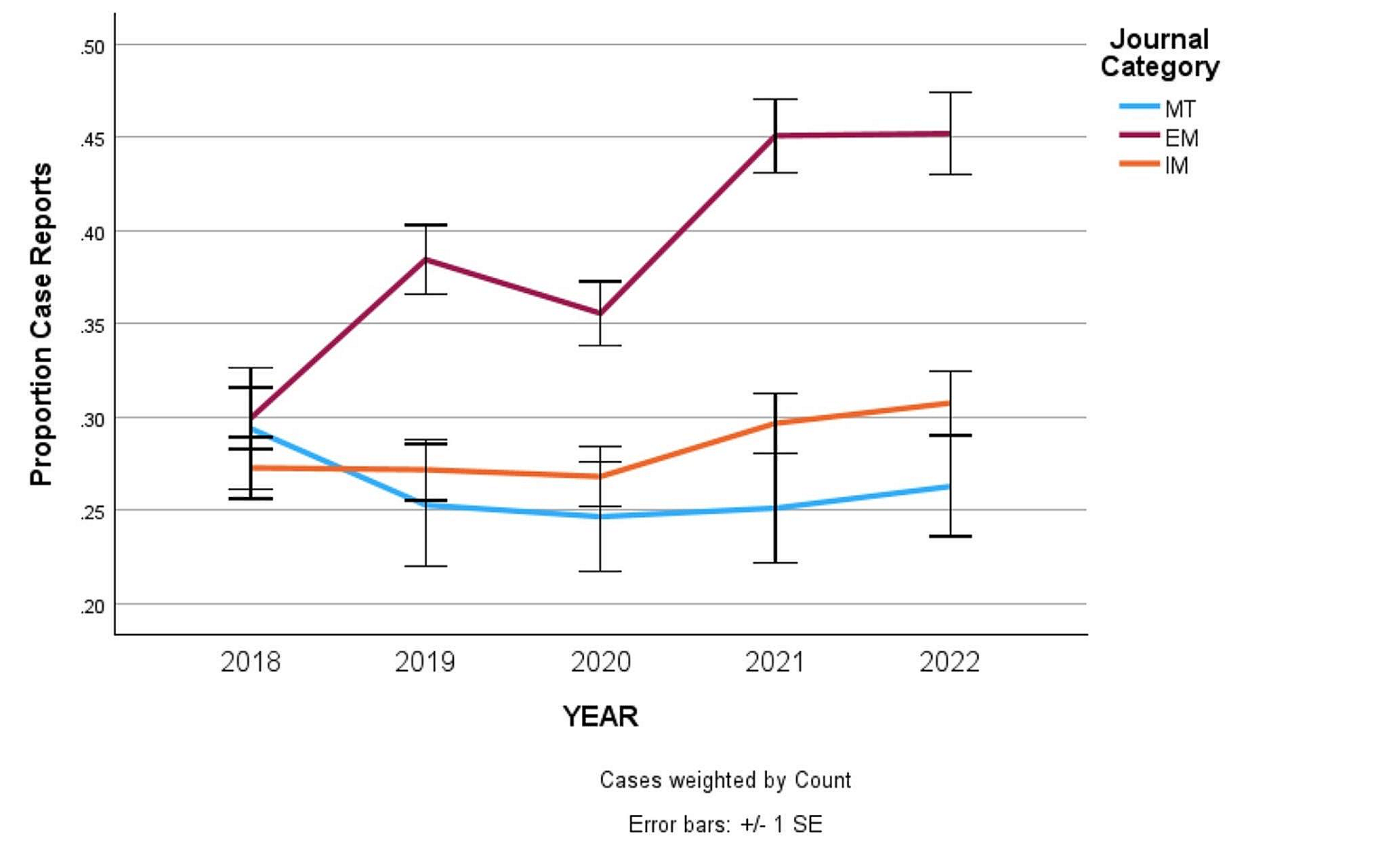
Chart of case report proportion of all articles for each year for each specialty. Pearson Chi-square analysis demonstrates significance between EM vs. MT and EM vs. IM for all years except 2018 (*p* < 0.002). No difference was found between MT and IM for any year

The percentage of cumulative case reports for each year was consistently greater in EM and IM than it was in the MT journals. Comparing the journal categories by year using a Pearson Chi-squares results in significance between EM vs. MT for all years except 2018 (*p* < 0.002), significance between EM and IM for all years except 2018 while no significance is found between MT and IM for any year. Logistic regressions results showed a strong linear trend in the proportion of case reports for EM *p* < 0.001, while no significant trend, linear or otherwise is noted for MT or IM, (*p* > 0.05 for both). Logistic regression also showed significant time by journal category interaction also indicating the trend in EM differs significantly from that of IM or MT. (Fig. 2).

## Discussion

Throughout history, case reports have been a foundational means of written story-telling to illustrate and disseminate information on illness and injury and discuss new and novel approaches of care and management of medical issues. They are found in the Edwin Smith Papyrus (16th to 17th dynasty), and feature prominently in the writings of Hippocrates (400 BCE) and Galen (200 CE) [2]. Up until the 1980’s case reports featured prominently in published literature compared with research articles [8]; this ratio reversed over the following years [9].

In the more modern evidence-based research epoch, case reports are criticized as containing information that is considered trivial with conclusions that are not supported and presented in an unstructured manner. They are considered part of the ‘lowest’ level in the research evidence hierarchy with respect to causality [10]. A review of published case reports trends in the psychiatry literature in the 1980s and 1990s describes the significant intentional decline in their number—from 17.4 to 2.4% due to factors including changes in focus and values applied to different types of research/methodology [9]; case reports are also infrequently cited by other articles, so in addition to their lower significance, journals may intentionally diminish the number of case reports they publish to protect their annual impact factors [10–12]. Lancet created a specific section for publication of case reports to distinguish it from the core research sections in 1995 [13]; other journals publish them primarily within the ‘correspondence’ section, with a similar goal.

Within medical toxicology, it has been introspectively stated, “With so few trials to enlighten our practice, the evidence we often find ourselves turning to is the lowly case report.” [14].

There are circumstances when case reports are particularly indispensable such as when disseminating information about events or occurrences that are critically important for clinicians to be aware of but rarely present; they are especially valuable for events that could not ever be ethically researched; they might inspire additional investigation into factors involved in the survival of a patient after a fatal exposure (e.g. a rare physiologic attribute or therapeutic combination). They are also often a list of ‘firsts’ that may call attention to something new or emerging —the first face transplant [15], the first patient with AIDS, the first patients with Covid-19, the first report of a deadly side effect of a new drug (e.g. thalidomide [16], etc.).

While case reports are used in every medical specialty, their attributes could not be more relevant than when characterizing a specialty like medical toxicology. MT is filled with events that are often: rare, fatal or near-fatal, happen to patients at high risk for bad outcomes; and where unique, exceptional events are often the rule, not the exception. Additionally, case reports may be pivotal to common MT practice (e.g. avoidance of treating an antimuscarinic tricyclic overdose patient with physostigmine [17] or avoidance of treating hyperkalemic digoxin toxic patients with intravenous calcium [18] -- whether they are foundationally true or not). Whatever way case reports are regarded, they often form the basis for additional work: including “higher value” publications that expand or investigate issues and themes initially presented in them. Efforts have been made to expand their inclusion in systemic reviews in circumstance where other literature availability is limited [19].

The limitations of this research paper include that the 8,246 articles were extracted by the same reviewer (JBH). While this adds the potential for bias, it limits variance of opinion or variety of interpretation for the data accumulation phase of the present work (which article belongs were). The definitions of the three categories of articles (Case reports, Research and Analysis articles, Excluded articles) were made to allow standardization while being broad enough to capture the variety of article types across the different specialties—future research may use alternate definitions for article types. There are potential limitations of the journals themselves: it is possible that printed journal articles differ from the publisher’s on-line version for each issue. While there are only a few broadly read MT journals, there are many IM and EM journals, and a different selection of journals might produce different results; additionally these journals may also discourage or encourage submission of case reports from authors either in print or through editorial bias and rejection resulting in different publication numbers—these factors are not included and could not be analyzed in our study. Although the present work was the first to review every article in each journal (and not an extrapolation [7, 9]) it is possible articles were missed.

## Conclusion

In the past 5 years, medical toxicology journals do not appear to have a higher prevalence of case reports than leading EM or IM journals. Additionally, MT journals demonstrate a declining trend in publishing case reports. Future research will have to be done to determine the effect on MT practice resulting from the diminished body of case report literature.
